# Multimodality imaging for transcatheter tricuspid valve repair and replacement

**DOI:** 10.3389/fcvm.2023.1171968

**Published:** 2023-07-12

**Authors:** Stephen Tomlinson, Carlos Godoy Rivas, Vratika Agarwal, Mark Lebehn, Rebecca T. Hahn

**Affiliations:** Cardiology Department, New York-Presbyterian/Columbia University Medical Center, New York, NY, United States

**Keywords:** multi-modality imaging, tricuspid regurgitation, transcatheter intervention, tricuspid valve, tricuspid incompetence

## Abstract

Transcatheter tricuspid intervention is a rapidly evolving field with multiple classes of therapeutic devices currently in development. Procedural success in tricuspid intervention is predicated on appropriate device selection for patient specific anatomy and satisfactory imaging for intra-procedural guidance. This review will outline protocols and methodology for multi-modality imaging assessment of the tricuspid valve and associated structures, with emphasis on anatomic and functional characteristics that determine suitability for each class of tricuspid intervention. Intra-procedural imaging requirements for each class of device, with design and procedural imaging guidance of specific devices, will also be addressed.

## Introduction

**Figure 1:**
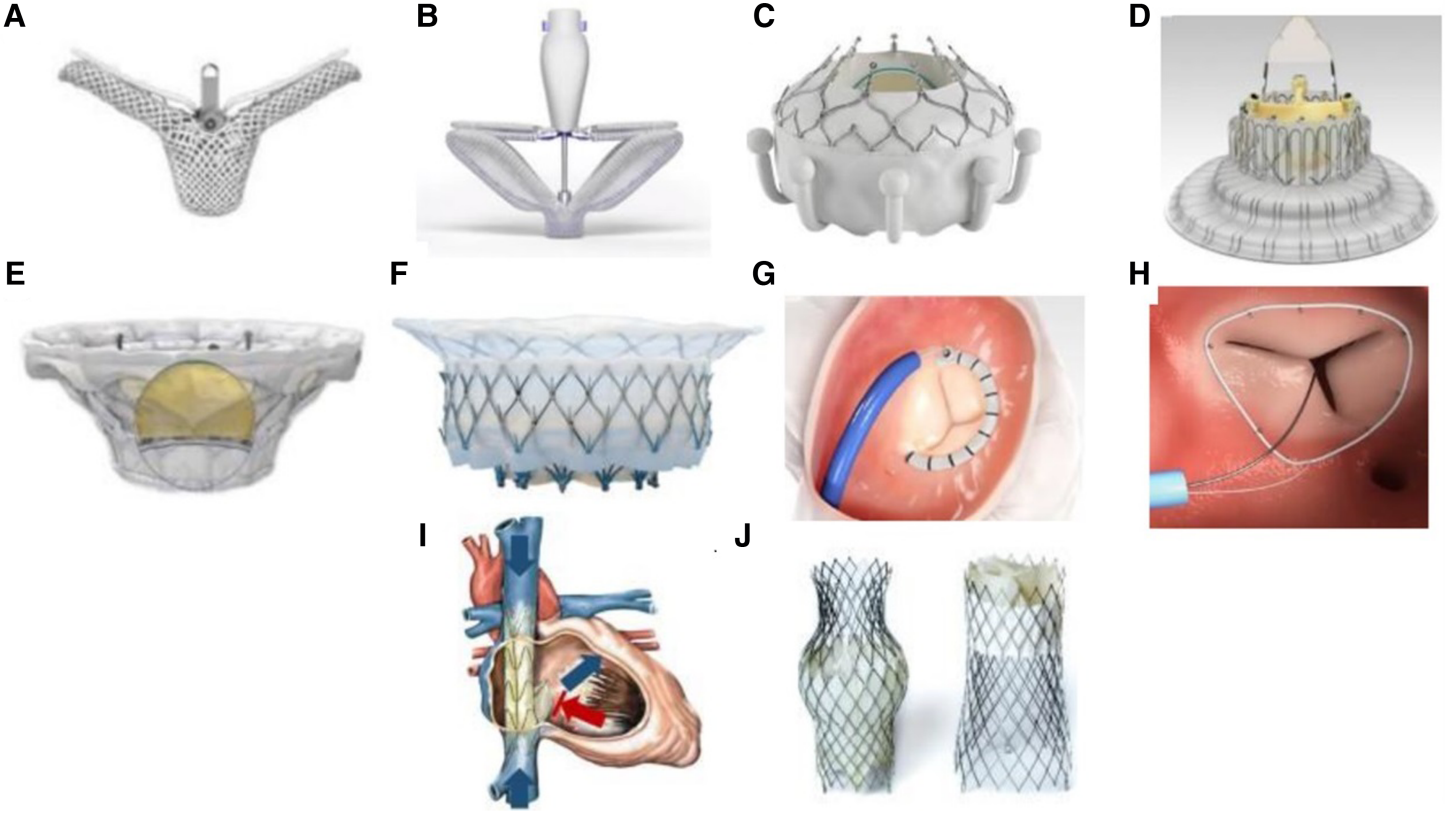
**Transcatheter Tricuspid Valve Devices**. (**A**) TriClip (Abbott Vascular, Santa Clara, California, USA); (**B**) PASCAL system (Edwards Lifesciences, Irvine, California, USA); (**C**) EVOQUE system (Edwards Lifesciences, Irvine, California, USA); (**D**) LuX-Valve (Jenscare Biotechnology Co., Ningbo, China); (**E**) Cardiovalve (Boston Medical, Shrewsbury, MA, USA); (**F**) Intrepid valve (Medtronic Plc, Minneapolis, MN, USA); (**G**) Cardioband tricuspid valve reconstruction system (Edwards Lifesciences, Irvine, California, USA); (**H**) Tri-Ring annuloplasty system (Cardiac implants, California, USA; (**I**) TRICENTO system (Medira AG, Balingen, Germany); (**J**) TricValve (NVT, Muri, Switzerland).

Tricuspid regurgitation (TR) is associated with increased mortality even after adjustment for left heart disease (1, 2). Surgical treatment of isolated TR is associated with excessive early mortality related to late referral and greater numbers of co-morbidities (3–6). In this setting, multiple transcatheter therapies for TR are currently being developed which may be associated with lower early morbidity and mortality (7–9). Therapies currently under investigation include devices for leaflet approximation, annuloplasty, orthotopic, and heterotopic valve replacement (Figure 1). Early feasibility investigations of these devices demonstrate their efficacy is governed by the underlying tricuspid valve (TV) anatomy and severity of TR (10). Imaging of the TV, therefore, is the cornerstone for transcatheter device selection, with device implantation dependent on imaging guidance for procedural success. This review will outline essential TV anatomy and multimodality imaging to guide transcatheter device selection and will outline intra-procedural imaging guidance of selected devices.

## Essential tricuspid valve anatomy

**Figure 2:**
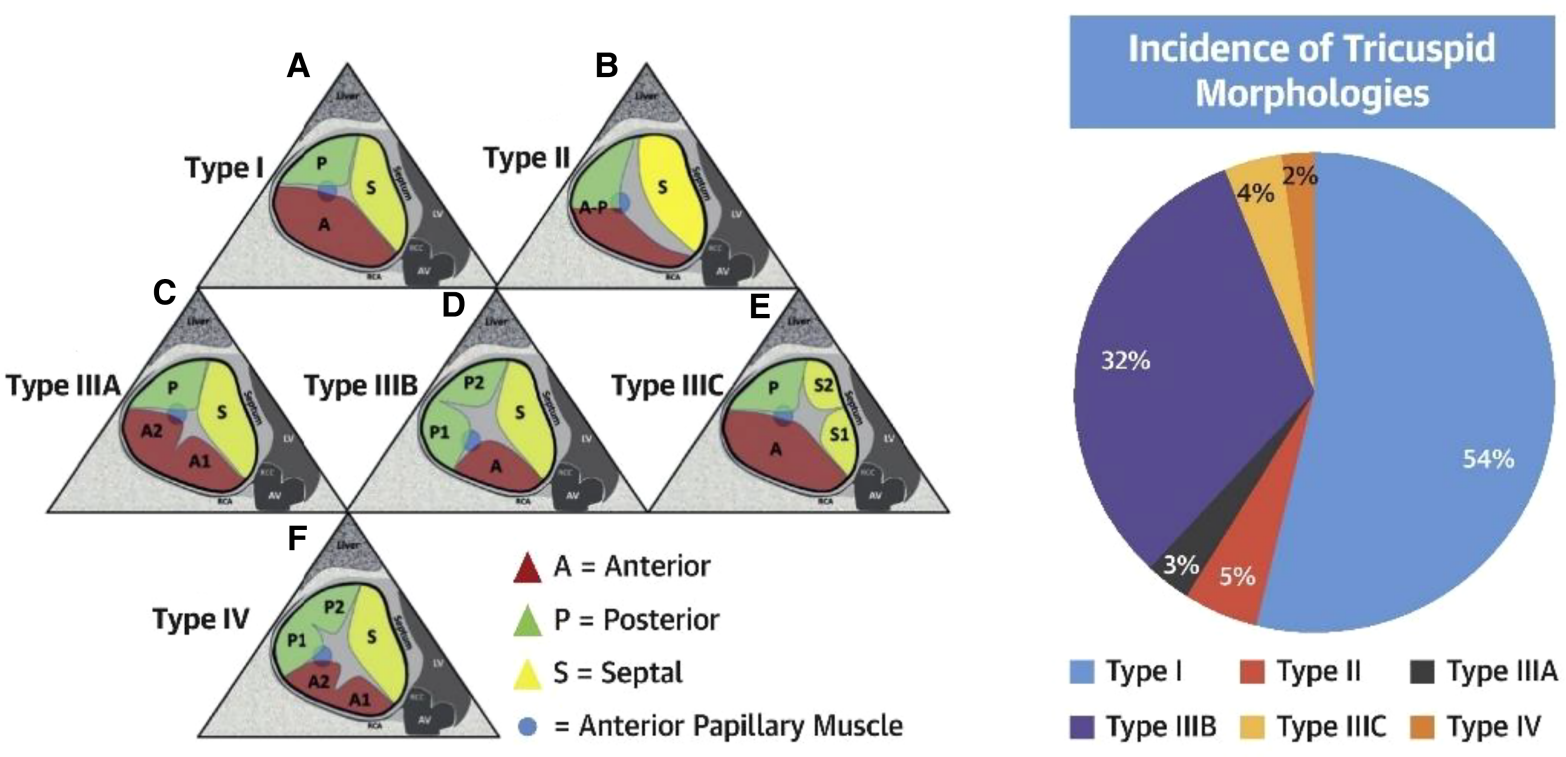
Tricuspid valve nomenclature classification scheme (reproduced with permission from (14)).

The TV is the largest cardiac valve with normal function dependent on the leaflets, annulus, papillary muscles, and chordae (11, 12). The TV leaflets are variable in size and number (13, 14), however, normally possesses three distinct leaflets commonly named the anterior, posterior and septal leaflets. The anterior and septal leaflets typically are the largest circumferentially, with a smaller posterior leaflet. Four-leaflet morphologies are the most commonly occurring variants (14, 15) (Figure 2). The leaflets are supported by primary and secondary chordae attached to the papillary muscles, and tertiary chordae attached directly to the septum. A recent proposal for standardization of multi-leaflet nomenclature suggests identifying the 3 primary leaflets using anatomic landmarks: (1) anteroseptal commissure adjacent to aortic valve from which the numbering of the multiple leaflets begins; (2) the interventricular septum which identifies the septal leaflet(s) and (3) the anterior papillary muscle (identified as the most anterior prominent papillary muscle, typically fused with the moderator band) which separates the anterior and posterior leaflets. The commissure between the septal and posterior leaflets is adjacent to the coronary sinus.

Identification of the anterior papillary muscle can be performed by size (largest), location on the anterolateral right ventricle (RV) wall, and incorporation into the moderator band. RV dilatation, particularly of the mid-ventricular free wall, may thus cause the papillary muscle to be more apically positioned, stretching the chordal attachments to the anterior and posterior leaflets, and causing tethering of the leaflets in systole. A variable number of posterior papillary muscles send chordal attachments to the posterior leaflet(s) and posterior segment of the septal leaflet. The septal and anterior leaflets also receive chordal attachments from septal papillary muscle(s) or from direct septal chordal attachments. Changes in position of the interventricular septum may affect the systolic mobility of the septal leaflet.

The normal TV annulus is a saddle-shape, ovoid structure (16, 17) which is nearly devoid of continuous collagen fibers. The space between the right atria and ventricles is composed of adipose tissue only on the anterosuperior and inferior sides. There is no adipose tissue on the septal side (18). Dynamic changes in annular size occurs throughout the cardiac cycle with the largest area found at end diastole (17). In the setting of secondary TR, the annulus becomes more circular and planar (19). Because the annulus is affixed to the fibrous cardiac skeleton along the septum, annular dilation occurs along the anterior and posterior leaflet attachments with sparing of the septal annulus (20). The regurgitation in functional TR is therefore typically along the length of the septal leaflet resulting in an elliptical or crescentic-shaped regurgitant orifice, with the greatest length in anterior-posterior direction (21).

## Classification of tricuspid regurgitation

**Figure 3:**
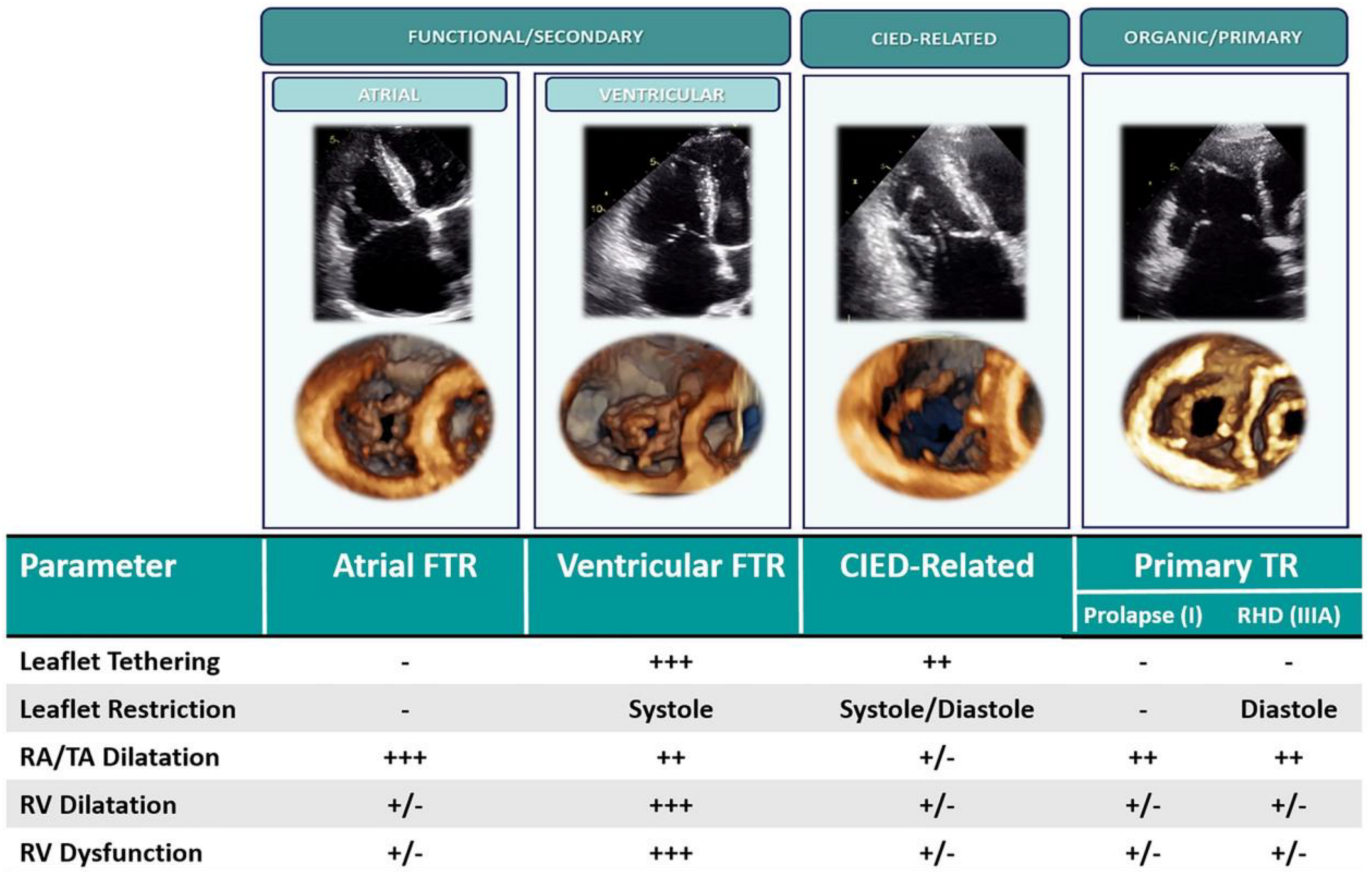
Classification of tricuspid regurgitation (16). CIED, cardiac implantable electronic device; FTR, functional tricuspid regurgitation; TR, tricuspid regurgitation; RA, right atrium; TA, tricuspid annulus; RHD, rheumatic heart disease.

TR classification can be established from morphological and functional assessment of the tricuspid leaflets, right atrium (RA), and RV, and is an important characterization that guides TTVI device selection. TR may be classified as primary, secondary or cardiac implantable electronic device (CIED)-related (Table 1 and Figure 3) (22, 23). Primary TR involves abnormalities of the tricuspid leaflets and encompasses fibroelastic degeneration, endocarditis, rheumatic disease, and carcinoid disease. Device therapy is guided by case-specific anatomical characteristics. CIED-TR may arise from primary device interaction with tricuspid leaflets or subvalvular apparatus, or secondary to pacemaker-induced RV dysfunction and dilation. Cases of CIED-TR with significant leaflet impingement are unfavorable for annuloplasty or leaflet approximation (24). Not all CIEDs, however, cause TR, and a comprehensive imaging assessment, typically using 2D and 3D echocardiography and computed tomography (CT) is required (25, 26).

**Table 1:** Pathophysiological classification of tricuspid regurgitation (23).

	Leaflet morphology	Pathophysiology	Etiology	Imaging
Primary	Abnormal	Loss of leaflet coaptation due to intrinsic changes, excessive mobility, or perforation	Myxomatous disease Endocarditis Trauma Carcinoid Rheumatic Iatrogenic (biopsy) Congenital	According to the etiology Description of the etiology, lesions and dysfunction
Secondary: Atrial	Normal	RA enlargement and dysfunction leading to TA dilation, conical remodeling of the RV	Atrial fibrillation	Severe RA remodeling RV basal diameter may be enlarged despite usually normal RV volume Leaflet tethering is absent or limited
Secondary: Ventricular	Considered normal	RV enlargement and/or dysfunction leading to significant leaflet tethering and TA dilation	Pulmonary hypertension RV cardiomyopathy RV infarction	Dominant mechanism is leaflet tethering ± TA dilation
CIED-related: Primary	Abnormal	Leaflet impingement Leaflet/chordal entanglement Leaflet adherence Leaflet laceration/perforation Leaflet avulsion (post lead extraction)	Pacemaker Implantable cardiac defibrillator Cardiac resynchronization therapy	3D echocardiograhy (±color) is mandatory for reliable diagnosis
CIED-related: Secondary	Normal	RV enlargement and/or dyssynchrony/dysfunction due to pace-maker stimulation and leading to significant leaflet tethering and TA dilation	Pacemaker rhythm	Dominant mechanism is leaflet tethering ± TA dilation

RA, right atrial; TA, tricuspid annulus; RV, right ventricle; CIED, cardiac implantable electronic device; 3D, Three dimensional.

Secondary TR (STR) accounts for >85% of cases of severe TR and may be atrial or ventricular in origin. Atrial secondary TR is characterized by right atrial and tricuspid annular dilation resulting in a mismatch between tricuspid annulus and tricuspid leaflet area. Atrial STR is typically observed in patients with chronic atrial fibrillation (AF). RV basal dilation and “conical” RV morphology is observed in these patients; however, leaflet tethering is rare until late in the disease (16, 27). Formal definitions of atrial STR are lacking, although the 2021 ACC/AHA guidelines characterize “isolated” or atrial STR as associated with AF, left ventricular (LV) ejection fraction >60%, pulmonary artery systolic pressure <50 mmHg and no left-sided valve disease, with normal-appearing TV leaflets (28). More recently, a clustering approach has been used to define atrial STR as TV tenting height ≤10 mm, midventricular right ventricular diameter ≤38 mm, and LV ejection fraction ≥50% (29). Atrial STR may be amenable to any class of TR therapy in the absence of prohibitive annular dilation or leaflet coaptation defect.

Ventricular STR is characterized by “spherical” mid-apical RV dilation with apical papillary muscle displacement and leaflet malcoaptation from tethering. Ventricular STR is typically observed in the setting of pulmonary hypertension (16, 27). Right atrial and tricuspid annular dilation may also arise in these patients from remodelling secondary to TR. Severe TV leaflet tethering is a strong predictor of recurrent TR following surgical repair (30) and thus ventricular STR patient anatomy is typically unfavorable for transcatheter annuloplasty devices. Depending on the severity of tethering and the coaptation gap, ventricular STR patients may not be ideal candidates for tricuspid transcatheter edge-to-edge repair (TEER) (10).

## Tricuspid valve multimodality imaging

### Echocardiography

A comprehensive evaluation of the TV should be performed by transthoracic (TTE) as well as transesophageal echocardiography (TEE). Adjunctive imaging tools include echo-fluoro fusion and 3D intracardiac echocardiography (ICE). TEE should be performed in all patients under consideration for transcatheter TV intervention (TTVI) to establish the severity and classification of TR, assess leaflet morphology and characteristics, TR jet size and location, and annular size. These anatomic parameters may permit patient-specific optimal device choice. Intra-procedural TEE with live three-dimensional (3D) multi-planar reconstruction (MPR) is critical for pre-procedural imaging (31) as well as imaging guidance of TTVI (32).

#### Transesophageal imaging protocol

**Figure 4:**
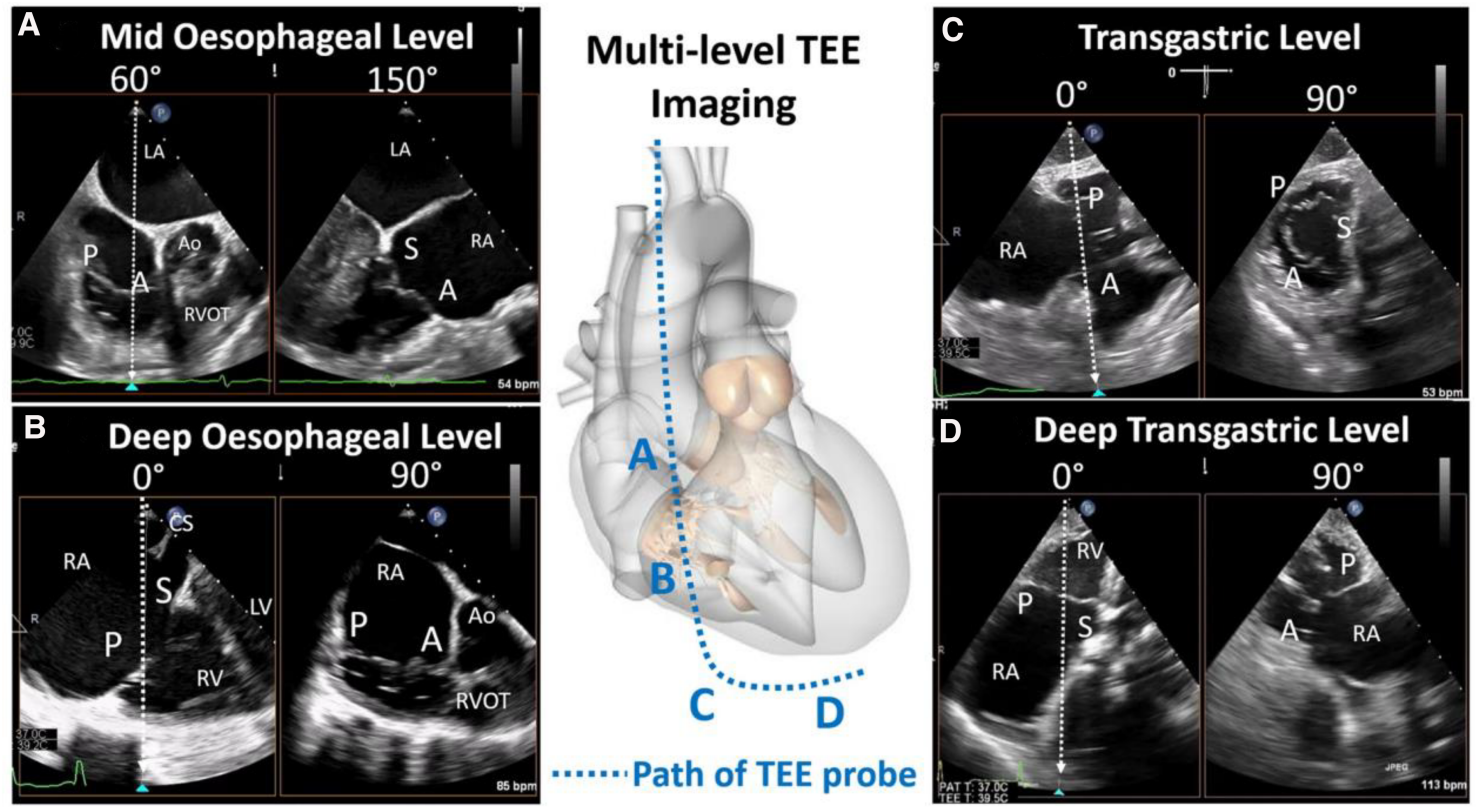
Transesophageal echocardiography imaging levels (reproduced with permission from (32)). Comprehensive tricuspid assessment requires imaging from multiple esophageal and gastric windows: (**A**) mid esophageal level, (**B**) deep esophageal level, (**C**) transgastric, and (**D**) deep transgastric. A, anterior leaflet; Ao, aorta; LA, left atrium; LV, left ventricle; P, posterior leaflet; RA, right atrium; RV, right ventricle; RVOT, right ventricular outflow tract; S, septal leaflet.

TEE assessment of the TV is complicated by the position and characteristics of the valve. As the largest and most anterior of the cardiac valves, the posterior position of the TEE probe requires far field imaging with acoustic shadowing from the interatrial septum or intervening left heart structures, as well as tangential imaging of the annular plane which relies on more limited lateral resolution. In addition, large 3D volumes must be obtained to encompass the entire valve and adjacent structures. To overcome these limitations, comprehensive TEE examination of the TV requires dedicated imaging from multiple esophageal and gastric levels. A detailed imaging protocol for tricuspid assessment is outlined in the American Society of Echocardiography guidelines, which, in brief, recommend multiplanar imaging from; the mid-esophageal window to evaluate the TV and RV in relation to surrounding anatomic structures, the distal esophageal window to eliminate intervening left-sided cardiac structures from view and optimize visualization of the tricuspid leaflets, the transgastric level for 2D/3D en face imaging of the TV and chordae attachments, and the deep transgastric window for both leaflet/subvalvular imaging and optimization of color and spectral evaluation of TR jets (Figure 4) (33).

#### 3D imaging and multi-planar reconstruction (MPR)

3D rendering of the tricuspid valve allows for rapid morphologic assessment and anatomic orientation. The use of advanced 3D manipulation with MPR of the 3D volume, is crucial for pre-procedural quantitative assessment of tricuspid leaflets and annular sizing (typically performed offline), as well as intra-procedural guidance of TTVI performed real-time.

Acquisition of 3D volumes should be performed from any imaging level with high quality 2D imaging. The acquired volume should include surrounding anatomical structures, ideally the aortic valve, in order to assist in 3D orientation (31). Once generated, the 3D rendered volume may be oriented to visualize an *en face* view of the TV. There are 2 common orientations of this *en face* view; the surgical perspective which rotates the image to place the aortic valve in the near field at ∼11 o'clock, or a non-rotated view where the aortic valve is at 5 o’clock (similar to a transgastric short-axis view).

For quantitation assessment of the tricuspid valve, a 3D volume can be manipulated off-line using MPR. Measurement of annular area, perimeter and diameters, as well as quantitation of leaflet lengths and coaptation gaps are required for determining anatomic feasibility of device placement. For procedural guidance, real-time multiplanar imaging using MPR allows continuous imaging of four different views: two orthogonal 2-D long-axis views with the tricuspid annulus perpendicular to the ultrasound beam, a 2D short-axis view at any level of the tricuspid valve apparatus, and a 3D rendered image. The most useful 2D long-axis views are typically the inflow-outflow view imaging the anterior leaflet to the right, and posterior leaflet to the left of the sector, and the orthogonal 4-chamber view with “lateral” leaflet to the right, and septal to the left of the sector. The “lateral” leaflet can be either the anterior or the posterior leaflet depending on the where the orthogonal plane is placed on the inflow-outflow view. The short-axis view can be positioned at any level, from the narrow apex to the broad base of the volume, depending on the requirements of device positioning and anchoring; imaging the annular level is most useful for annuloplasty devices and orthotopic valve replacements whereas at the leaflet level may be optimal for TEER devices. The 3D rendered image can be defined by the user as any of the three 2-D images in the MPR screen but most often the short-axis 3D rendering is preferred for TEER devices to orient the device arms and for orthotopic valve replacements for positioning in the annulus. Importantly, all planes can be manipulated throughout the procedure to inform the proceduralist of device positioning relative to the tricuspid valve and surrounding anatomy.

#### TR severity quantitation and grading

Quantitation of TR severity is necessary to establish pre-procedural baseline and inform prognosis. Contemporary classification of TR severity involves a five-grade scale, extending the traditional “mild”, “moderate” and “severe” with “massive” and “torrential” grades, with severity established through a combination of quantitative parameters (Table 2) (34). Methodology for TR quantitation has been extensively described elsewhere (21). Early feasibility studies of TTVI showed a reduced ability to achieve residual TR ≤2+ with massive and torrential baseline TR (35). Patients with massive or torrential TR have increased risk for all-cause mortality and rehospitalization for heart failure 1 year compared to those with severe disease, even after successful TTVI (36). Procedural success, defined as residual TR 2+, was also achieved less frequently in patients with baseline massive or torrential TR, but is associated with improved clinical outcome at 1 year, irrespective of the baseline severity of disease. The TRILUMINATE Pivotal study which used the new five-grade TR severity scale, showed a graded response of TR reduction and improvement in quality of life (37). Together these studies justify the current European guidelines recommendation to use of the 5-grade scale for patients considered for TTVI (23, 38).

**Table 2:** Contemporary grading of tricuspid regurgitation severity (34).

Variable	Mild	Moderate	Severe	Massive	Torrential
VC (biplane)	<3 mm	3–6.9 mm	7–13 mm	14–20 mm	≥ 21 mm
EROA (PISA)	<20 mm^2^	20–39 mm^2^	40–59 mm^2^	60–70 mm^2^	≥ 80 mm^2^
3D VCA or quantitative EROA			75–94 mm^2^	95–114 mm^2^	≥ 115 mm^2^

VC, vena contracta; EROA, effective regurgitant orifice area; 3D VCA, three-dimensional vena contracta area.

#### Tricuspid morphology and leaflet assessment

**Figure 5:**
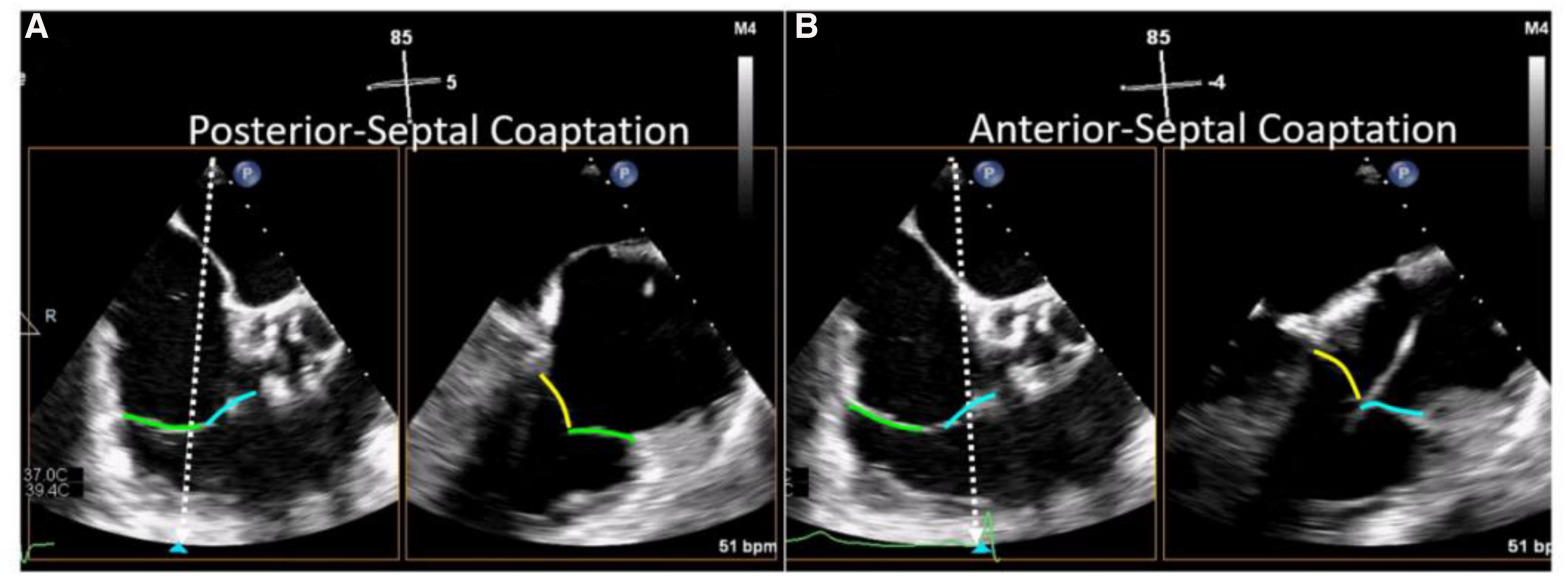
Biplane TEE Imaging of the tricuspid valve (reproduced with permission from (32)). The mid-esophageal right ventricular inflow-outflow view (at ∼50–80°) is considered the TV 'commissural' view with the anterior (blue line) and posterior (green line) leaflets imaged and the septal leaflet (yellow line) behind the imaging plane. Moving the orthogonal biplane cursor towards the posterior wall (**A**) images the posterior and septal leaflets. Moving the orthogonal biplane cursor towards the aorta (**B**) images the anterior leaflet near the aorta and the septal leaflet.

Evaluation of TV morphology and leaflet assessment is crucial to inform transcatheter device selection and should be systemically evaluated in all pre-procedural TEE examinations. A useful technique for leaflet assessment is biplane imaging from the mid or distal esophageal inflow-outflow view (also referred to as the “commissural view”) of the TV, which is typically acquired at 50–80 degrees of mechanical rotation. Using simultaneous biplane imaging, sweeping the orthogonal imaging plane from posterior (left side of sector) to anterior (right side of sector, near the aortic valve) visualizes the coaptation of the posterior-septal, then anterior-septal commissures, respectively (Figure 5). In this manner, specific leaflet abnormalities as well as coaptation gaps, can be localized and measured. Leaflet characteristics of relevance when planning TTVI include leaflet length, tethering, and coaptation defect width.

Transgastric imaging facilitates evaluation of TV morphology and further localization of the origin of TR. From the mid-gastric window, right flexion and anteflexion of the TEE probe produces a right ventricular inflow view with the tricuspid annulus parallel to the insonation beam. Biplane imaging through the tricuspid annulus then produces an en face view of the TV (Figure 4). The en face image can also be obtained from single plane imaging by straight anteflexion in the mid gastric window with mechanical rotation set at 30–60 degrees. En face tricuspid imaging facilitates assessment of tricuspid morphology, which has been recently highlighted as heterogenous and complex (14). Evaluation of tricuspid leaflet morphology is relevant in pre-procedural assessment as increasingly complex leaflet morphologies may be unfavorable for procedural efficacy of TEER (39, 40). Importantly, coaptation gaps should only be measured from the transgastric 2D short-axis views if the leaflet tips are imaged; studies suggest that this view frequently overestimates these gaps compared to the RV inflow-outflow views (41). TV morphology and leaflet characterization may thus be more accurately assessed with 3D echocardiography and multi-planar reconstruction.

#### Right heart size and function

RV function is an important determinant of patient outcome following TTVI. Assessment of RV function may also serve as a useful clinical tool to guide timing and risk stratification of TTVI. Because longitudinal shortening is a dominant mechanism of RV ejection, tricuspid annular plane systolic excursion (TAPSE) as well as tissue Doppler s' and RV free wall strain are important echocardiographic measures to assess RV function (42). Reduction in TAPSE is a known predictor of increased mortality following TV surgery (43). Analysis from the TriValve registry similarly demonstrates reduced survival at one year in patients with TAPSE < 17 mm undergoing TTVI compared to those with preserved RV function (36).

Propensity matched analysis from the TriValve registry suggests the degree of RV dysfunction in patients undergoing TTVI may be an important parameter influencing post-procedural outcomes. In the TriValve registry, TTVI in patients with mid-range reduced TAPSE of 13–17 mm was associated with survival advantage compared to conservative therapy at one year. In contrast, TTVI in patients with TAPSE < 13 mm conferred no survival advantage (44). The absence of survival advantage in this group suggests that adverse remodelling in advanced RV dysfunction may be irreversible, and intervention in this group may be “futile” from a prognostic perspective. Patients with advanced RV dysfunction may also lack the reserve to compensate for the acute increase in afterload observed following significant reduction in TR, with a rare case of shock and RV failure reported following successful TTVI (45).

TAPSE has numerous limitations for RV assessment, being dependent on Doppler alignment for accuracy and non-reflective of radial RV contraction. Alternate markers of RV function may better predict outcomes and guide selection for TTVI. In a prospective cohort of 75 patients undergoing tricuspid TEER in which all patients had 3D RV ejection fraction at baseline, 3D RV ejection fraction of <44.6% predicted mortality at one year, whereas TAPSE did not (46). RV free wall longitudinal strain and RV ejection fraction assessed by CMR are also of interest, having been demonstrated as independent predictors of mortality in patients with severe TR (47, 48). RV ejection fraction ≤45% measured by CMR (49) or 3D echo (46) in patients undergoing transcatheter tricuspid valve repair is an independent predictor for the composite end-point of all-cause mortality and HF hospitalization.

Isolated assessment of RV function without reference to ventricular loading conditions may not be ideal for patient selection in TTVI, as it fails to assess RV compensation to specific loading conditions or ventricular contractile reserve. The assessment of right ventricular-pulmonary artery (RV-PA) coupling reflects the efficiency with which RV stroke work is transferred into the PA and can be calculated echocardiographically as the ratio of TAPSE: pulmonary artery systolic pressure (PASP). In compensated states, increase in RV afterload from rising PASP is associated with a proportionate increase in RV contractile function, reflected by TAPSE. As the RV decompensates, however, there is uncoupling of afterload and RV contraction, and the RV-PA ratio declines. In a large study of medically managed patients with TR, a TAPSE/PASP value <0.31 mm/mm Hg was used to define RV−PA uncoupling and to dichotomize the population (50). RV-PA uncoupling was the only echocardiographic parameter independently associated with all-cause mortality (HR 1.462; 95% CI, 1.192 to 1.793; *p* < 0.001) after correcting for potential confounders. Recent analysis of a TTVI registry demonstrates that a low RV-PA ratio of <0.406 at baseline is a powerful predictor of post-procedure shock and of all-cause mortality at one year following TTVI, and may be a robust tool to guide patient selection and procedural timing (51).

#### Annular sizing

**Figure 6:**
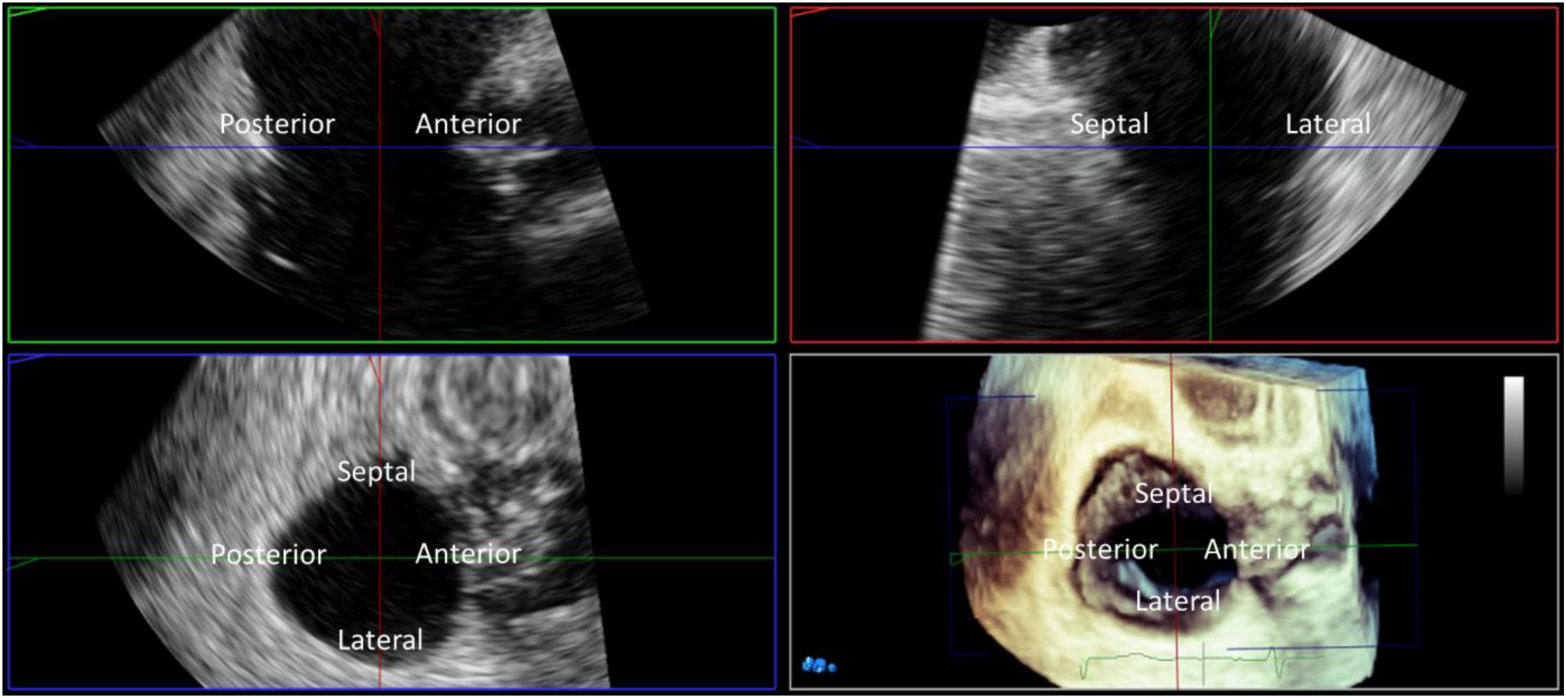
Three-dimensional (3D) TEE multiplanar reconstruction. 3D imaging with multiplanar reconstruction of the tricuspid valve from the mid esophageal window in mid diastole. The 3D block is presented in the ‘non rotated’ view with the reconstructed long axis 2D images displayed in the familiar ‘commissural’ and corresponding septo-lateral orthogonal image. Direct measurement of tricuspid annular dimensions is performed from planimetry of the 2D short axis image aligned with the tricuspid annulus.

Annular area by TEE can be estimated from 2D imaging by incorporating the anterior-posterior and septal-lateral annular diameters into the formula for an ellipse, or measured from 3D volumes (Figure 6). Two methods have been correlated with CT; direct annular area and perimeter planimetry of the tricuspid annulus from 3D multi-planar reconstruction of the short-axis annular imaging plane, or using in-direct measurement adapting the advanced mitral annular quantitation program (52). The in-direct measurement of the tricuspid annulus correlates best with CT measurements and may be used for device sizing, however direct planimetry of the annular area allows for a more accurate quantitation of diastolic stroke volume.

## Computed tomography

CT facilitates anatomical assessment of the TV complex, RV, and associated vascular structures. CT is requisite in all TTVI planning, excluding TEER. CT may also play an adjunctive role in evaluating TR severity and RV function in cases of suboptimal echocardiographic assessment.

### Image acquisition protocol

Multiphasic electrocardiogram (ECG)-gated acquisition should cover the entirety of the cardiac cycle. This can be performed using ECG-triggered prospective acquisition or retrospective helical scanning; the latter preferred if cine images are required (53). More advanced scanners with higher temporal resolution and a greater number of detector rows allow greater craniocaudal coverage and faster acquisitions with shorter breath-hold time and reduced contrast volume (21).

Image acquisition may be challenging due to the presence of AF or intracardiac devices, both of which are frequently concurrent with TR. In the case of AF or other tachyarrhythmia, the use of retrospective ECG-gated protocols is preferentially used, albeit at a higher radiation dose (54).

Clear visualisation of the right heart and TV requires a dedicated CT protocol to ensure adequate contrast opacification around the TV annulus and minimise artefacts. Intravenous injection of a standard non-ionic contrast agent with an iodine concentration of 270 to 370 mg/ml is used. A triphasic protocol (i.e., 60%/40% contrast/saline mixture at a rate of 4 ml/s, followed by a 25%/75% contrast mixture at a rate of 4 ml/s, and concluded with a 20 ml normal saline push at 4 ml/s) (21) yields optimum results with decreased risk of premature triggering, minimization of contrast media, reduction of streak artefact within the SVC, and decreased inhomogeneous attenuation of the right atrium, compared to biphasic administration (55).

### TR severity

Direct quantification of TR severity using cardiac CT is not widely established. CT features suggestive of TR include RA and RV dilation, reflux of contrast media into the inferior vena cava (IVC), and dilated hepatic veins. Opacification of the hepatic veins and IVC on first-pass arterial phase CT imaging is suggestive of TR with high specificity and sensitivity (21). Other anatomical surrogates of TR include the anatomic regurgitant orifice area (AROA), tricuspid annulus area, and tricuspid leaflet tethering.

The AROA is a novel marker of TR severity which measures the defect area in tricuspid leaflet coaptation. AROA is measured dring mid systole (20%–30% RR-interval) via planimetry of the minimum area bordered by the tricuspid leaflet tips in the short-axis view defined by double-oblique multiplanar reconstruction (21). Recent investigation demonstrates AROA has a strong correlation with 3D vena contracta area (VCA) assessed by TEE (56).

### Tricuspid leaflet tethering area and height

Tricuspid leaflet tethering area and height are assessed on the long-axis two- or four-chamber view images by measuring the distance between the annular plane and leaflet coaptation point, and by tracing the leaflets from the annular plane, respectively (55). Tricuspid leaflet tethering height assessed by CT correlates with echocardiographic assessment of TR severity. Severity of tricuspid leaflet tethering assessed by CT has also been demonstrated as an independent predictor of TR recurrence following surgical annuloplasty procedures (57).

### Tricuspid annulus area

The tricuspid annulus area is measured from reconstructed short-axis imaging obtained at mid diastole (60%–80% of the RR interval) with manual selection of the plane at the level of the TV annulus on the four-chamber and two-chamber views. The normal maximum annulus diameter is 3.0–3.5 cm on the short-axis image. Functional TR is characterised by annular dilation >4 cm (54).

### RV size and function

RV morphology assessment is required for planning orthotopic TV replacement. Necessary assessment includes measurement of RV length, assessed in the four-chamber view at end systole as the distance between the tricuspid annulus and perpendicular RV apex, and evaluation of anatomic structures including papillary muscles and the moderator band, within this path (58). RV ejection fraction assessed by CT is accurate and reproducible compared to cardiac magnetic resonance (CMR) imaging as the reference method. RV ejection fraction is calculated from reconstructed short-axis views tracing the inner RV myocardial border at end diastole and end systole. Myocardial trabeculations and papillary muscles are generally included in the tracing as they can be excluded from volumetric quantification using attenuation-threshold based selection. RV volumes and ejection fraction are calculated using Simpson's method (55).

### Localization of the right coronary artery

Localization of the course of the right coronary artery (RCA) is crucial in assessment of TTVI to evaluate risk of coronary impingement during implantation of the annular anchoring systems. MPR of the two-, four-chamber, and short-axis views of the TV during mid diastole are evaluated to measure the distance between the RCA and the annulus. Unfavorable course of the RCA is described as <2 mm distance to the annulus and occurs most commonly in association with the posterior TV leaflet given its position along the diaphragm (58).

### Access evaluation

Evaluation for TTVI requires assessment of central and peripheral venous anatomy for device delivery. Device specific evaluation may include assessment of femoral or jugular vein diameters, cavo-atrial angulation, or detailed evaluation of caval anatomy. Orthotopic tricuspid valve replacement typically requires large bore peripheral venous access. Orthotopic tricuspid valve replacement and TEER are complicated by acute cavo-atrial angulation (10, 59). Heterotopic tricuspid valve replacements have specific caval anatomical requirements, outlined below (60, 61).

## Cardiac magnetic resonance imaging

CMR is not routinely indicated for pre procedural evaluation of TTVI, however, may be used in an adjunctive role to guide patient selection through assessment of right ventricular function and TR severity. Right ventricular image acquisition is performed with cine steady-state free-precession sequencing. Volumetric analysis is performed from stacked short-axis cine images from the base to the apex of the right ventricle, obtained separately using ECG-gated acquisitions. The endocardial margin of each slice is traced at both end-diastole and end-systole. Post-processing software is used to calculate the RV volume, mass, and ejection fraction (55). CMR is considered the gold standard for right ventricular volumetric analysis. Semi-quantitative assessment of TR severity is performed by assessment of local signal drop out area related to flow turbulence/acceleration on steady state free precession cine imaging (21). Quantitative assessment of TR can be assessed with multiple techniques. Indirect calculation of TR volume is performed by subtraction of left or right ventricular forward stroke volume from right ventricular volumetric stroke volume assessed by geometric analysis. Left and right ventricular forward stroke volume can be directly assessed by velocity encoded imaging from short axis imaging with a defined area of interest aligned perpendicular to the direction of flow at the level of the pulmonic or aortic valve. Direct assessment of TR volume by velocity encoded imaging, however, is complicated by the non-planar saddle shaped tricuspid annulus and significant motion of the annulus throughout the cardiac cycle and is not widely validated (21). Recent investigation has highlighted the potential role of 4D flow velocity encoded imaging to overcome these limitations, however, this technique remains largely investigational at this time. The role of CMR for assessment of tricuspid leaflet morphology is not well established.

## Fluoroscopy

Fluoroscopic projections of the right ventricular long axis perpendicular to tricuspid annulus and of the en face tricuspid annulus should be established during TTVI for assessment of device positioning and axiality with the tricuspid annulus. These projections may be derived from the pre-procedural CT and are typically the right anterior oblique cranial (RAO/CRA) and left anterior oblique caudal (LAU/CAU) views, respectively (62, 63). Roadmaps from caval, right ventricular, and right coronary angiography are useful tools to identify anatomical landmarks and guide TTVI. Hybrid imaging with anatomical landmarks derived from CT or 3D echocardiography overlaid on fluoroscopic projections has been described for procedural guidance in a number of TTVI case reports, however, as of yet, is not established as a routine tool for guidance of TTVI (64, 65).

## Intracardiac echocardiography

Two-dimensional ICE has been used as an adjunct to TEE imaging for TTVI (66, 67). Three-dimensional ICE probes have recently been introduced which, although lacking the field of view and 3D spatial and temporal resolution of TEE, have also been used as an adjunctive imaging tool for TTVI (68). ICE catheter positioning in the right atrium near the tricuspid annulus provides near field, high spatial resolution imaging of regions of the TV. ICE, therefore, may potentially be a useful adjunctive modality for TTVI guidance when TEE imaging is suboptimal. In TEER, in particular, ICE may be utilized to optimize imaging of the TEER device arms, leaflet grasp within the device arms, tissue bridge generation, and reduction in TR, enabling procedural success in the presence of suboptimal TEE imaging.

## TTVI therapies

**Figure 7:**
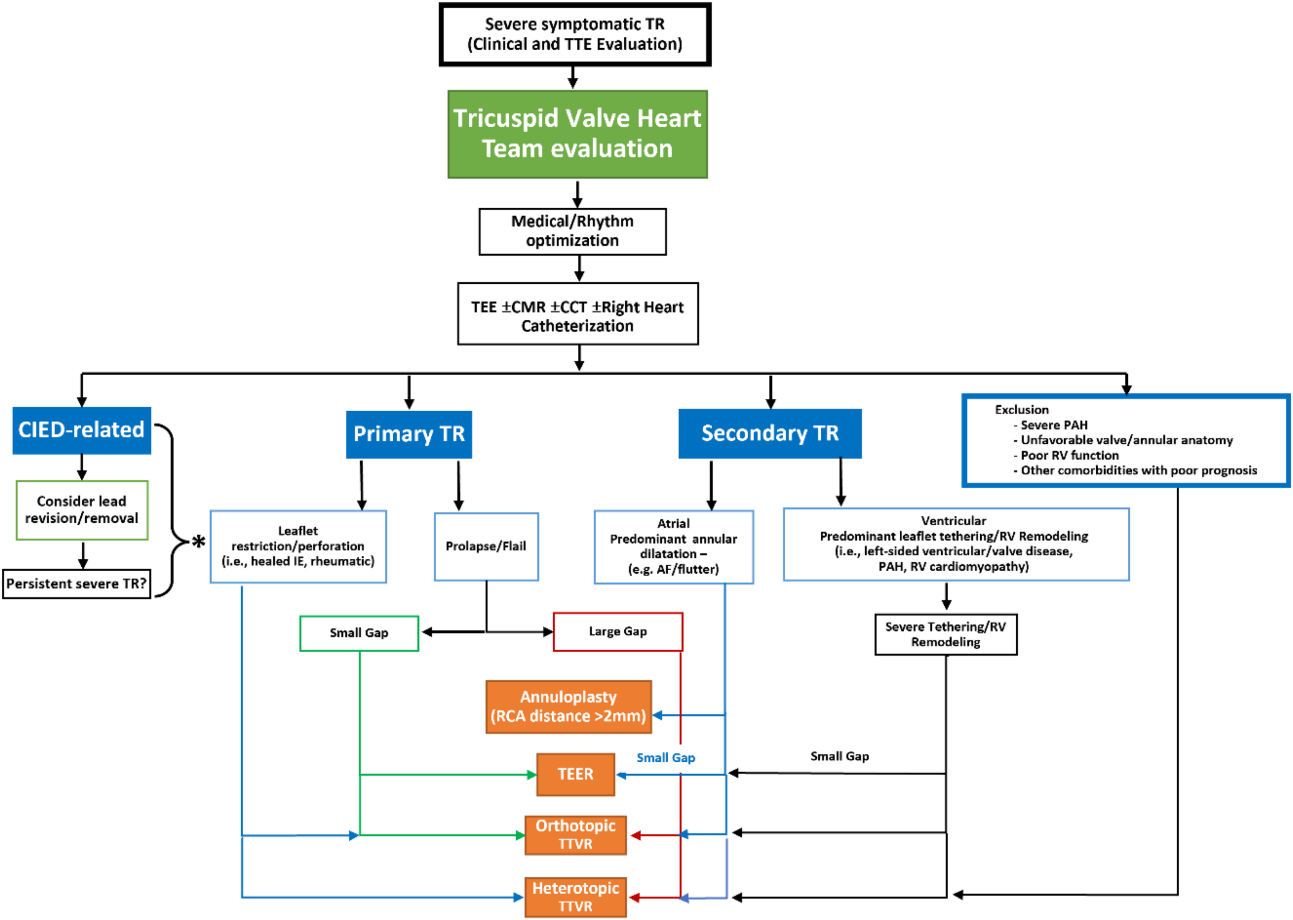
Proposed workflow algorithm for device class selection for transcatheter tricuspid valve intervention (reproduced with permission from (93)). TEE, transesophageal echocardiogram; CMR, cardiac magnetic resonance imaging; CCT, cardiac computed tomography; CIED, cardiac implantable electronic devices; TR, tricuspid regurgitation; PAH, pulmonary arterial hypertension; RV, right ventricular; IE, infective endocarditis; AF, atrial fibrillation; RCA, right coronary artery; TEER, transcatheter edge to edge repair; TTVR, transcatheter tricuspid valve replacement.

A number of recent single-site surgical reports suggest with careful patient selection, advanced surgical techniques and periprocedural management, isolated TV surgery can be performed with lower morbidity and mortality (5, 69). However, the patient populations in these studies do not resemble the patients now being referred for possible TTVI, being much younger (mean age ∼56 years) with indications for surgery that include endocarditis and congenital disease. A current isolated tricuspid valve surgery risk score reported the median in-hospital mortality for functional disease was ∼10% (70). Thus transcatheter therapies continue to be enthusiastically explored (10). A proposed workflow algorithm for device selection in Figure 7 is based on anatomic and morphologic characteristics of TR. Intra-procedural imaging requirements for each class of device, with design and procedural overview of specific devices, is outlined below.

### Leaflet approximation

**Figure 8:**
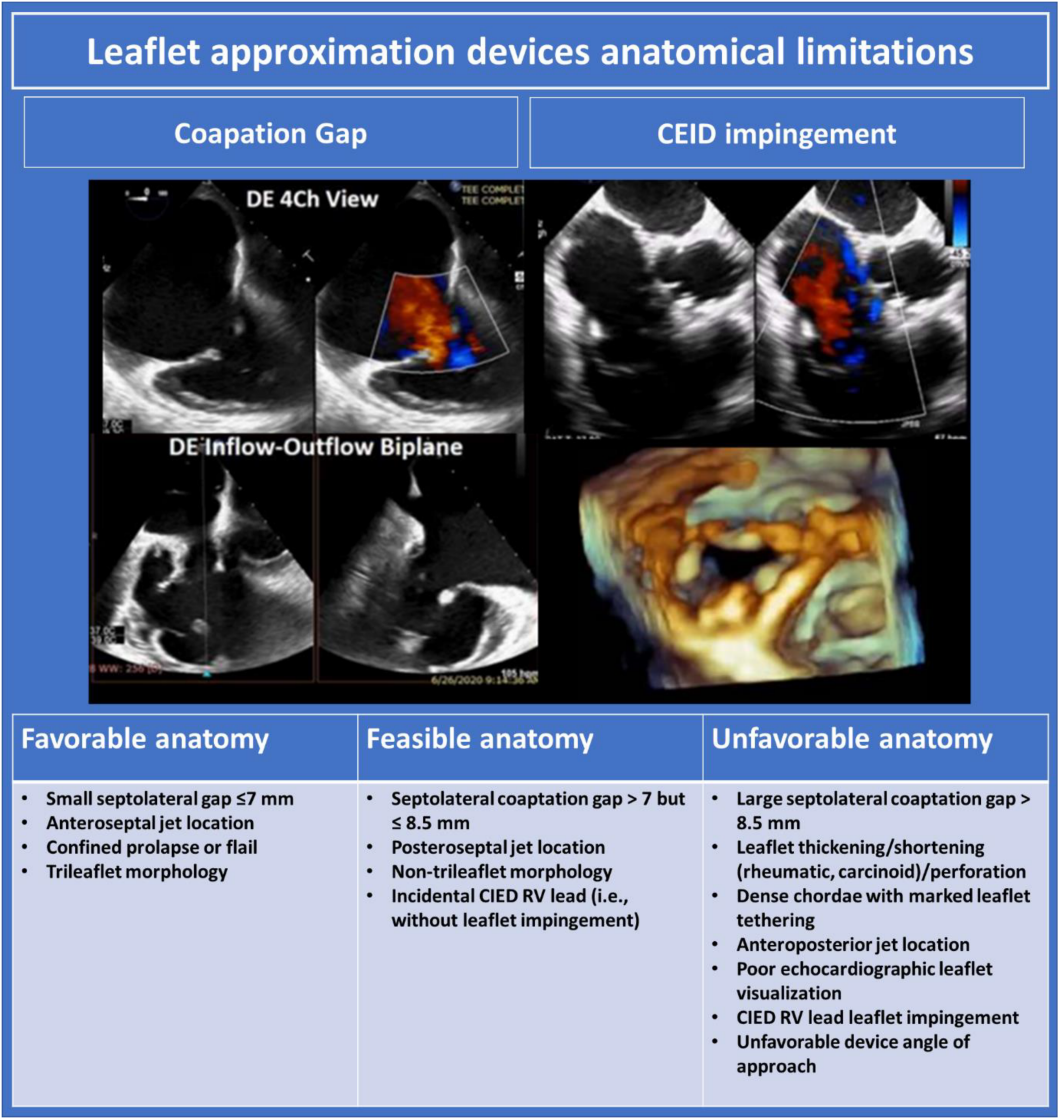
Leaflet approximation devices anatomical limitations (10). DE, deep esophageal; TR, tricuspid regurgitation; CIED, cardiac implantable electronic devices; RV, right ventricle.

Leaflet approximation is most commonly performed via TEER. Other novel devices for enhanced leaflet coaptation currently under investigation are considered under “miscellaneous therapies” later in this review. Anatomic determinants of suitability for leaflet approximation devices are outlined in Figure 8. Two TEER devices, the TriClip (Abbott Vascular, Santa Clara, California, USA), and PASCAL system (Edwards Lifesciences, Irvine, California, USA) have received CE Mark and are commercially available in Europe. Both devices have demonstrated significant reduction in TR severity with excellent safety (35, 71–73). Design characteristics of the two devices are outlined below. Steps for pre-procedural and intra-procedural imaging are considered together.

#### Triclip

The TriClip system consists of a steerable guide catheter and clip delivery system including an implantable clip. The clip is composed of two clip arms and grippers with vertically arrayed retention elements. Four clip sizes are available: the NT and NTW, featuring 9 m clip arms with 4 and 6 mm widths, respectively, and the XT and XTW, featuring 12 mm clip arms and 4 and 6 mm clip widths, respectively. The grippers can be actuated simultaneously or independently. Results from the TRILUMINATE Pivotal Trial which randomized patients with severe, symptomatic TR to TriClip or medical therapy, confirmed the safety of the device and showed a significantly improved patient quality of life at one year, but failed to show an improvement in mortality or heart failure hospitalizations compared to medical therapy (37). This may be related to low event rates in both cohorts and the short follow-up period. Patients in the trial will be followed for 5 years however cross-overs at 1 year may influence the ability to test this theory.

#### PASCAL system

The PASCAL repair system consists of a guide sheath, steerable catheter, and implant catheter. The device implant is composed of a central spacer designed to reduce leaflet stress with adjacent paddles and clasps. The clasps have a horizontal row of four retention elements at their tip to enhance leaflet capture. Two sizes are available: the PASCAL Ace, with 6 mm wide and 15 mm long paddles, and the PASCAL, with 10 mm wide and 16 mm long paddles. The clasps can be actuated simultaneously or independently. Both devices can be elongated when positioning and manoeuvring to reduce risk of chordal entrapment. The randomized controlled CLASP II TR trial (ClinicalTrials.gov Identifier: NCT04097145) is currently enrolling with a 2-year composite endpoint.

#### Pre-procedural imaging for TEER

TEER relies on feasible leaflet anatomy and TEE image quality for procedural success. Cardiac CT and CMR are typically not required in pre-procedural evaluation.

Pre-procedural TEE should be performed with the patient in minimal lateral positioning, or, if tolerated, supine, to replicate the conditions available for intra-procedural imaging. For procedural success in TEER, adequate tricuspid leaflet visualisation must be available from at least one esophageal level and from the transgastric level, although guidance of TEER may be feasible with sub-optimal esophageal windows if 3D ICE is available. TEE assessment should be performed as per protocol outlined above. Anatomic characteristics influencing favourability for TEER are outlined in Figure 8.

#### Intra-procedural imaging guidance for TEER

Procedural assessment should begin with full comprehensive TEE examination including formal quantitation of TR severity while under anaesthesia to establish a baseline for post-procedural comparison.

Procedural guidance begins as the device delivery system enters the RA from the IVC. Imaging at this stage is necessary to assess device trajectory and ensure the device does not damage the intra-atrial septum, or interact significantly with the Eustachian valve, Chiari network, pacing hardware, or other RA structures which may restrict movement of the device. Imaging is obtained by acquiring a bicaval view from the mid-esophageal window before lowering the mechanical rotation and retro-flexing the probe until visualization of the IVC and RA junction is optimized. Once the device is located, live 3D or biplane imaging should be employed to track the device relative to the tricuspid annuls as it is manoeuvred superiorly to the mid-atrial level. Once the device has reached mid RA level, flexion of the delivery catheter will lower the device towards the tricuspid annulus. This may be tracked by anti-clockwise rotation of the TEE probe, bringing the TV into view. The imager should then pause, optimize imaging windows, and evaluate the optimum location for TEER.

The optimal location for TEER may be denoted by specific anatomic characteristics, such as the location of a flail segment, or the location at which colour Doppler suggests the TR is most severe. In the absence of a specific anatomic defect, or in cases where the TR is diffuse, anterior-septal TEER may be the most effective initial step in reducing TR (74). TEER registry data demonstrates the anterior-septal commissure is treated in 66% of cases, the posterior-septal commissure in 33% of cases, and the anterior-posterior commissure in 1% of cases (73). Two clips are required in 54% of cases, one clip required in 37% of cases, and three or more clips are rarely required.

Leaflet assessment at the site of intended site of TEER, and guidance of the device to this location, is most effectively performed with 3D MPR. To assess, the MPR cross hair in the *en face* image should be positioned at the intended site of repair and orientated at the intended angle of device deployment, which, to preserve leaflet geometry, should typically be perpendicular to the line of coaptation. Leaflet characteristics (i.e., leaflet length and coaptation gap) at the intended site of TEER will then be appreciable in the corresponding imaging plane. If characteristics at the initial site of assessment are unfavorable, dragging the perpendicular plane across the breadth of coaptation facilitates “scanning” of the tricuspid leaflets to identify a feasible site for repair.

Once the site for repair has been established, the device should be maneuvered to this location using the MPR crosshairs as a guide. The open device arms orientation should be aligned with the established MPR configuration at the optimum site for repair. Short-axis transgastric imaging can also be used to evaluate orientation. Independent grasping controls should be confirmed prior to advancing the device into the RV.

The device should then be advanced below the valve and orientation re-assessed. Two different methods can be used; (1) 3D volumetric imaging from esophageal levels of imaging, which typically requires reducing the 3D gain resulting in dropout of the thin TV leaflets and clear imaging of the device in the RV, and (2) 2D short-axis transgastric imaging of the leaflet tips to evaluate device orientation, with the caveat that off-axis imaging of the valve may lead to inaccurate position assessment. Once orientation is confirmed, the device is retracted toward the leaflet, avoiding chordal entanglement and visualizing leaflets laying flat (i.e., without curling) on the device arms/paddles.

Leaflet capture may be performed under 3D MPR or 2D single or biplane imaging. 3D MPR has some advantages to 2D imaging as it facilitates continuous surveillance of device orientation on short-axis or 3D *en face* views, while imaging trajectory and leaflet engagement in orthogonal 2D long-axis views. In some cases, however, 3D MPR resolution is inadequate to assess leaflet engagement. In such cases, 2D single plane (typically at 0–20° or ∼140–160° mechanical rotation), or biplane imaging using the commissural view as the primary image and the biplane marker directly through the device to display leaflet engagement in the orthogonal plane, may be used. With either technique, the imager should look to demonstrate leaflet tissue laying on the device base arms/paddles before leaflet capture is performed. Unlike TEER for the mitral valve, the “bouncing” of the grippers/clasps due to systolic closure of the captured native leaflets may not be seen on the low pressure right side. If repeated attempts at leaflet capture are unsuccessful, device rotation should be suspected, and orientation reassessed.

Post-deployment assessment includes evaluation of the severity of residual TR, tricuspid gradients and valve area, RV function, and hemodynamic benefits. TR quantitation following TEER is most accurately assessed by 3D planimetry of the vena contracta area, as the regurgitant orifice area by PISA may be distorted by the TEER device typically overestimating severity, and the diastolic stroke volume cannot be accurately measured for quantitative assessment given the distortion of the inflow by the device. Tricuspid gradients and valve area are rarely significantly elevated following deployment of a single device. RV function is typically unchanged unless there is a dramatic reduction in TR severity with an associated significant increase in effective RV afterload. Reduction in hepatic vein reversal, and increased RV and LV stroke volume, are indicative of successful repair.

## Orthotopic tricuspid valve replacement

**Figure 9:**
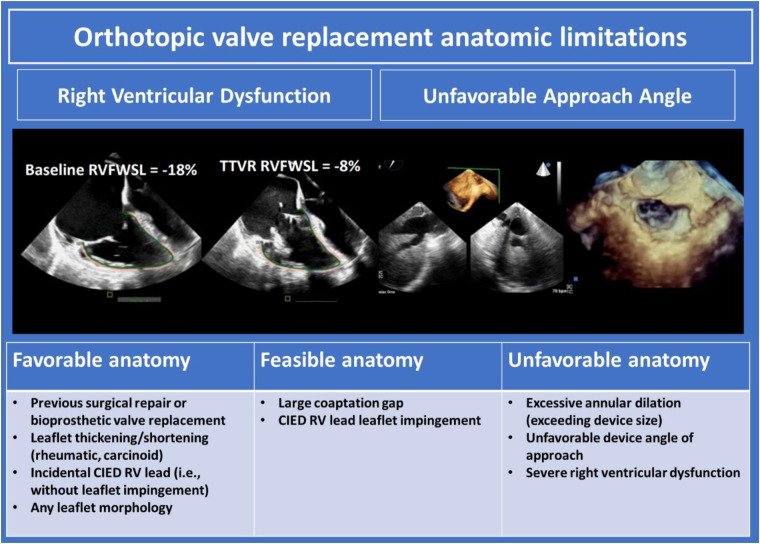
Orthotopic valve replacement anatomic limitations (10). CIED, cardiac implantable electronic devices; RV, right ventricle.

A number of orthotopic tricuspid replacement devices are currently in development. The devices rely on either radial force, tricuspid leaflet engagement, or septal insertion for implantation and stability. Anatomic determinants of suitability for orthotopic TV replacement are displayed in Figure 9, and include tricuspid annulus size, RV size and function, peripheral venous calibre, and cavo-atrial angulation. Leaflet characteristics are of secondary importance and typically will not inform device selection unless the mechanism of anchoring depends on intact leaflets. Valve implantation may be performed in the presence of CIED leads as long as the CIED interaction with tissue does not preclude accurate positioning of the device. Pre-procedural imaging must ensure the patient has adequate TEE imaging windows to facilitate intra-procedural guidance including confirmation of device sizing. CT facilitates other necessary facets of anatomic assessment. Intra-procedural imaging guidance relies on fluoroscopy and TEE to guide device advancement from the caval vessels through the RA and to the annular level. 3D MPR is then critical to evaluate device depth, location, and axiality with the tricuspid annulus.

### EVOQUE

The EVOQUE system (Edwards Lifesciences, Irvine, California, USA) consists of an implantable bioprosthetic valve, steerable delivery catheter, and dilator kit. The valve element is composed of a trileaflet bovine pericardial valve mounted on a self-expanding nitinol frame with a fabric skirt. There are nine anchors on the ventricular portion of the valve frame for native leaflet capture and device anchoring. The system is delivered via 28Fr femoral venous access and is available in three sizes: 44, 48, and 52 mm. Of the devices currently in trial or development, the EVOQUE system currently has the greatest body of clinical evidence, with results from the single arm TRISCEND study of 176 patients demonstrating excellent device efficacy and safety out to one year, including; survival of 90.1%, freedom from heart failure hospitalization at 88.4%, and significant and sustained TR reduction, with 97.6% of patients with mild or trace TR (8, 75–77). The pivotal TRISCEND II trial (NCT04482062), comparing optimal medical therapy plus TTVR with the EVOQUE system to optimal medical therapy alone is currently underway.

#### Intra-procedural imaging

Procedural imaging guidance is performed with fluoroscopy and TEE. The device is advanced via the delivery steerable catheter across the tricuspid annulus into the right ventricle. Device location, depth, and axiality are optimized with imaging. The device anchors are exposed through retraction of the delivery capsule and positioned below the tricuspid leaflets and above the papillary muscle heads. Further device expansion then positions the anchors below the annulus for native leaflet capture. Leaflet capture by all nine anchors is assessed by 3D MPR by rotation of the MPR lines on the short-axis image with the crosshair placed in the centre of the device at the level of the anchor tips, sequentially demonstrating leaflet engagement and annular position of each anchoring element on the long-axis orthogonal views. After satisfactory leaflet capture is confirmed, the device is fully expanded and released from the delivery system. Valve hemodynamics, valvular and paravalvular regurgitation are assessed as per standard protocols.

### Lux-Valve

The LuX-Valve (Jenscare Biotechnology Co., Ningbo, China) is a bioprosthetic valve and stent prosthesis composed of a trileaflet bovine pericardial valve, a self-expanding nitinol valve stent with an atrial disc, an interventricular septal anchoring element, and two clips for anterior leaflet attachment. The implant relies on the septal anchoring element and leaflet clips, rather than radial force, for attachment and stability. Early iterations of this device used minimally invasive right thoracotomy for transatrial access (78, 79). Recently, first in-human experience with the LuX-Valve Plus, delivered via right transjugular access, has been reported (80). The Lux Valve Plus is delivered via right jugular venous access through a 36Fr outer sheath containing the 33Fr delivery catheter. A minimum right jugular venous diameter of 10 mm is required for delivery. Three valve stent sizes of 40, 50 and 55 mm are available.

#### Intra-procedural imaging

Intra-procedural imaging is performed combining fluoroscopy and TEE to assesses device location, orientation, and trajectory. After access and introduction of the delivery system into the RV, the system is centred in the tricuspid annulus perpendicular to the annulus. The outer sheath is then withdrawn, releasing the valve stent, anchoring elements, and leaflet clips. The leaflet clips are then expanded and the system withdrawn towards the tricuspid annulus, facilitating leaflet clip capture of the anterior leaflet. The leaflet clips may be visualised to “swing” with the motion of the anterior leaflet on fluoroscopy or TEE when capture is successfully performed. After leaflet capture is confirmed, the atrial disc is released. Optimization of the orientation axiality of the implant within the tricuspid annulus is then performed. Once positioning is satisfactory, a nitinol anchor is fired from the anchoring element into the ventricular septum, securing and finalizing deployment of the valve. Following deployment, valve hemodynamics, valvular, and paravalvular leak are assessed as per standard protocols.

### Cardiovalve

The Cardiovalve (Boston Medical, Shrewsbury, MA, USA) is a trileaflet bovine pericardial valve mounted on a dual self-expanding nitinol frame composed of welded ventricular and atrial elements. The ventricular frame has 24 grasping legs for tricuspid leaflet engagement and the atrial frame has a dacron fabric covered flange for anchoring and sealing. The valve is delivered through 28Fr transfemoral access. Three sizes of 45, 50 and 55 mm are available (65, 81).

#### Intra-procedural imaging

The device is advanced via a steerable catheter to the level of the tricuspid annulus. Central annular location and annular axiality are confirmed via imaging. The grasping legs of the ventricular stent are then exposed, and the device advanced into the RV. The device is then withdrawn towards the RA to capture the native leaflets between the grasping legs and annulus. 3D MPR imaging is employed to ensure leaflet engagement by all grasping legs to achieve device stability and prevent paravalvular leak. 3D MPR assessment of grasping leg engagement is performed by placing the MPR crosshairs in the centre of the device at the level of the grasping leg tips and rotation of the MPR lines to sequentially assesses each grasping element. Device position and axiality is adjusted as required to achieve optimal leaflet capture. The atrial flange is then exposed, the valve fully exposed, and deployed.

### Intrepid valve

The Intrepid (Medtronic Plc, Minneapolis, MN, USA) system consists of a self-expanding implantable bioprosthesis, delivery system, and loading system. The bioprosthetic implant is composed of a 27 mm trileaflet bovine pericardial valve mounted on an inner nitinol stent, an outer nitinol fixation ring that engages the tricuspid annulus, and an echogenic woven polyester skirt that forms an atrial brim. Device fixation is achieved by small outer stent cleats that produce frictional engagement with the native valve leaflets, and by varying degrees of radial stiffness in the outer stent that create a cork-like confirmation effect around the tricuspid annulus. The device is available in 44 and 48 mm sizes and is delivered via femoral venous access through a 35 mm sheath. First in-human experience using the Intrepid valve for treatment of severe TR has been described and an early feasibility trial is currently underway (NCT04433065) (82–84).

#### Intra-procedural imaging

TEE and fluoroscopy are used to guide the delivery system to the tricuspid annulus and obtain central annular position perpendicular to the tricuspid annulus. Once satisfactory position has been achieved with the superior device capsule ∼2 cm above the tricuspid annulus, the system is pressurized to expand the atrial brim. Re-evaluation and positioning of the device guide by 3D MPR imaging and fluoroscopy is undertaken before the device is fully expanded and deployed. Valve hemodynamics, valvular and paravalvular regurgitation are assessed as per standard protocols.

## Percutaneous tricuspid annuloplasty

**Figure 10:**
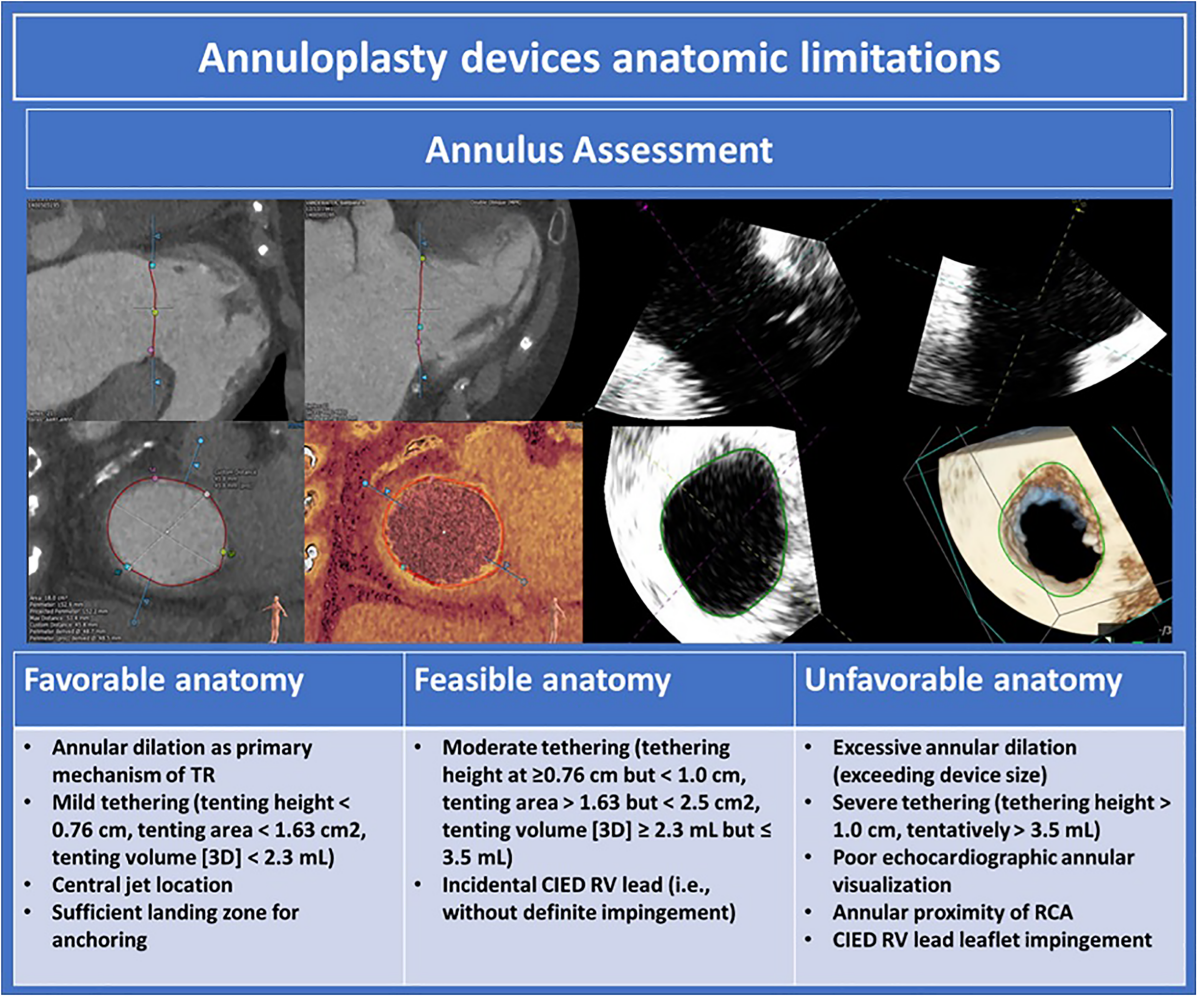
Annuloplasty devices anatomic limitations (10). TR, tricuspid regurgitation; CIED, cardiac implantable electronic devices; RCA, right coronary artery.

Percutaneous tricuspid annuloplasty devices reduce tricuspid annular diameter to improve leaflet coaptation and reduce TR. Annuloplasty procedures are best suited in cases of functional TR where annular dilation is the primary mechanism responsible for TR. Anatomic determinants of suitability for annuloplasty are outlined in Figure 10. Pre-procedural imaging requires TEE to exclude unfavorable leaflet characteristics such as severe tethering or CIED impingement. CT is crucial to assess annular size, annular shelf depth, annular tissue quality, including the presence of annular calcification, and relation of the RCA to the tricuspid annulus.

### Cardioband

The Cardioband tricuspid valve reconstruction system (Edwards Lifesciences, Irvine, California, USA) is a transcatheter tricuspid annuloplasty device for the treatment of severe functional TR. The original device used in the early feasibility trial was composed of a contraction wire within a polyester fabric covering and radiopaque markers. The device has since been iterated, however, the mechanics of device implantation remains similar. Up to 17 individual anchors (prior device) or 14 individual anchors (current device) are implanted in the annulus avoiding the region of the triangle of Koch. After implantation, the contraction wire is cinched to reduce annular diameter and reduce TR. Results from the Edwards Cardioband Tricuspid Valve Reconstruction System Early Feasibility Study of 37 patients demonstrate 73.0% of patients achieved ≤ moderate TR at one year, with significant reduction in tricuspid annular diameter and RV end diastolic volume (7, 85).

#### Intra-procedural imaging

Procedural imaging guidance is performed using fluoroscopy and TEE. RCA angiography is performed, and a guidewire is passed into the RCA, prior to commencing the procedure. Fluoroscopic projections of the RV long axis perpendicular to the tricuspid annular plane and of the *en face* tricuspid annulus are established to assess device trajectory and axiality with the annulus. The delivery system is advanced to the right atrium and position above the anterior-septal commissure (prior device) or posterior-septal commissure (current device) with position confirmed on 3D TEE. Each anchor is delivered just outside the leaflet hinge point and stability is confirmed with a push pull test assessed with fluoroscopy and TEE. After the final anchor is deployed, the Cardioband is then cinched to reduce annular diameter under continuous TEE assessment and adjusted to achieve the optimum hemodynamic result.

### Tri-Ring

The Tri-Ring annuloplasty system (Cardiac implants, California, USA) is an adjustable transcatheter annuloplasty ring implanted through a two-stage process. The ring is introduced via 22Fr transjugular access by a delivery system containing a balloon expandable scaffold. The ring is circumferentially attached to the tricuspid annulus by a series of small barbs. The ring is left in place for 90 days during which time tissue growth surrounding the barbs firmly secures the ring to the tricuspid annulus. The ring is then tightened using a 26Fr adjustment tool to reduce annular diameter and improve leaflet coaptation. First in-human experience with the Tri-Ring system has been described and the Early Feasibility Study of the Cardiac Implants Percutaneous Ring Annuloplasty System for the Treatment of Functional Tricuspid Regurgitation trial is currently underway (NCT04890821) (86).

#### Intra-procedural imaging

Procedural imaging guidance is performed with fluoroscopy and TEE. The delivery system is advanced to the RA and the ring expanded to a round circular shape via the balloon expandable delivery scaffold. The ring is maneuvered to the central annulus and annular axiality established. The ring is attached to the annulus via the anchoring barbs. The delivery the system is collapsed and removed from the body. A thin tether remains attached to the ring and is burrowed in a temporary pouch under the skin. After ∼90 days and adequate tissue growth has covered and stabilized the ring, the adjustment tool is advanced over the tether and pulled, cinching the tricuspid annulus. Cinching is performed under continuous TEE assessment to achieve an optimal hemodynamic result. Finally, the implant tether is secured, and the adjustment tool removed.

## Heterotopic TV replacement

**Figure 11:**
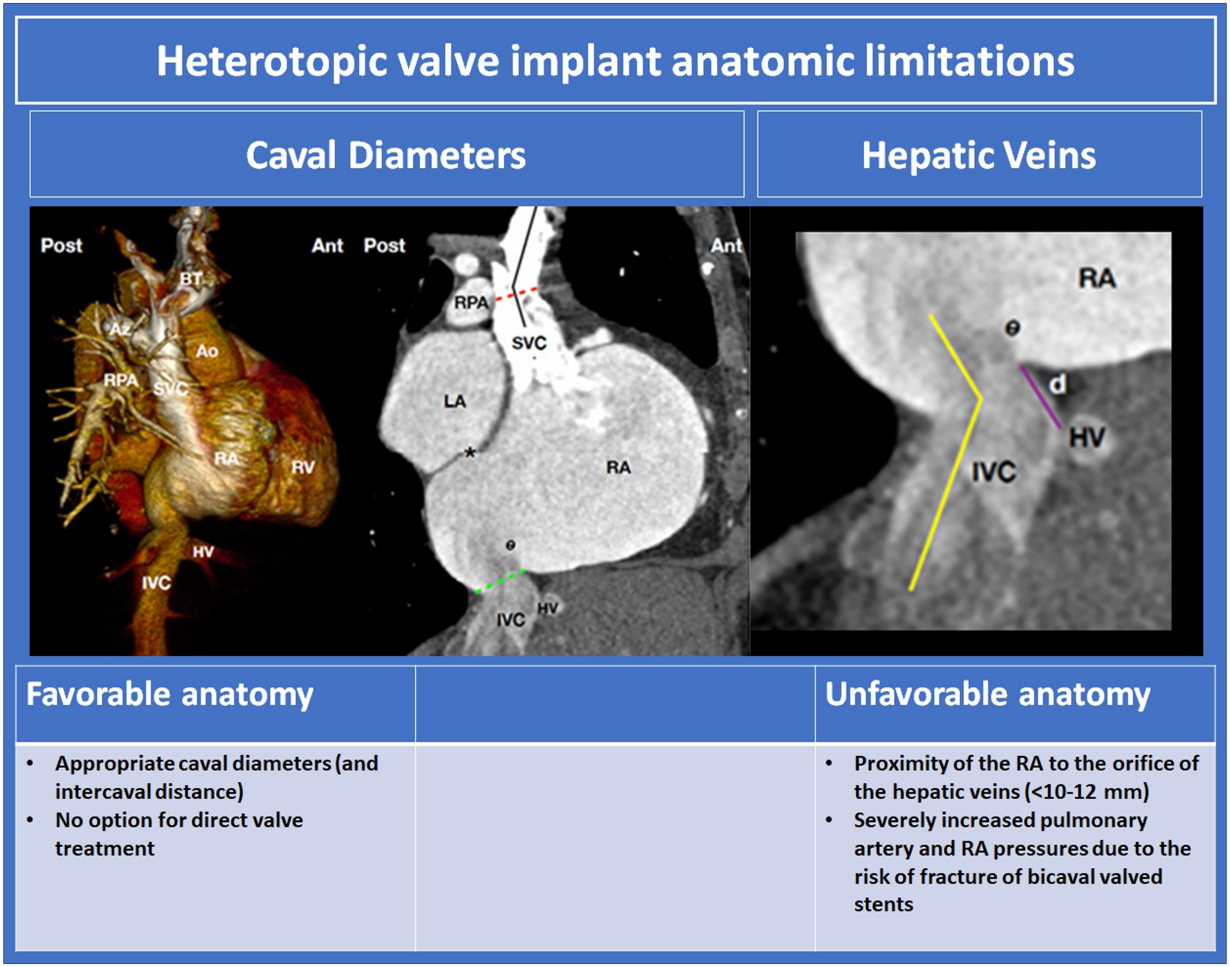
Heterotopic valve implant anatomic limitations (10, 60). BT, braciocephalic trunk; Az, azygos vein; Ao, aorta; RPA, right pulmonary arery; SVC, superior vena cava; RA, right atrium; RV, right ventricle; HV hepatic vein; IVC, inferior vena cava; SVC superior vena cava; LA left atrium.

Heterotopic TV replacement has been demonstrated to reduce symptoms of congestion in patients with symptomatic severe TR. The efficacy of this therapy for RV remodelling, however, is unclear. The anatomic determinants of suitability for this procedure are displayed in Figure 11. Pre-procedural assessment relies on CT for RA and caval analysis. Intra-procedural imaging guidance predominantly uses fluoroscopy.

### Tricvalve

The TricValve (NVT, Muri, Switzerland) system is a dedicated bicaval therapy for treatment of severe TR. The system comprises of two different, self-expanding nitinol stents harbouring pericardial leaflets, designed specifically for the anatomic characteristics of the superior vena cava (SVC) and IVC. The SVC stent is contoured with a high radial force “belly” and a long skirt for device fixation and prevention of paravalvular regurgitation. The IVC stent has high proximal radial force for stent fixation at the RA/IVC junction and a short skirt so as not to impede hepatic venous return. Both stents are delivered via femoral venous access using a 27.5Fr delivery system. Multiple sizes for the IVC and SVC stents are available. Results from the TRICUS-EURO study, a single arm observational study of 35 patients, demonstrate excellent procedural efficacy and safety, and significant improvement in quality of life and NYHA classification at 6 month follow up. No significant improvement in hemodynamic parameters or RV volumes, however, were observed following treatment (60, 87).

#### Pre-procedural imaging

Baseline transthoracic echocardiography (TTE) should be performed for screening of exclusion criteria (RV S' < 13 mm, RVSP > 65 mmHg). Device sizing and anatomical suitability is assessed with CT. For the SVC stent, evaluation of the diameter at the superior cavo-atrial junction, level of the PA, confluence of the innominate trunk, and distance between these points, is required. A minimum distance of 50 mm between the superior cavo-atrial junction and innominate confluence is required to accommodate the SVC stent. For the IVC stent, assessment of the inferior cavo-atrial junction, IVC diameters above and below the hepatic veins, and distance between the hepatic veins and RA is required. A short distance between the hepatic veins and RA and acute angulation of the cavo-atrial junction are unfavorable characteristics that may compromise device stability and predispose to paravalvular regurgitation.

#### Intra-procedural imaging

Deployment of the SVC stent is guided by fluoroscopy alone. After establishing femoral venous access, a Swan-Ganz catheter is placed in the right PA and a pigtail placed in the SVC. An SVC angiogram is performed to identify anatomical landmarks and establish a roadmap for deployment. The device is deployed from cranio-caudal direction. Ideal placement is with the broadest section of the “belly” at the level of the right PA catheter. For the IVC stent, a venogram of the IVC, including the inferior cavo-atrial juncture, serves as a guide for deployment. Ideal deployment of the IVC stent is with 5–12 mm of the proximal stent strut into the RA. TTE or TEE are useful adjuncts for IVC stent positioning as both readily image the inferior cavo-atrial junction and can be used to evaluate stent height and paravalvular regurgitation. Both the SVC and IVC stents are recapturable up to 80% deployment.

### TRICENTO

The TRICENTO system (Medira AG, Balingen, Germany) is a self-expanding bicaval stent graft for treatment of severe TR. The system is composed of a self-expanding nitinol frame lined with porcine pericardium with a lateral bicuspid valve that faces the RA and prevents systolic backflow. The device is delivered via the right femoral vein through a 24Fr sheath. The device is custom fabricated to individual patient specifications. Retrospective registry experience of 21 patients demonstrates excellent procedural safety and improved NYHA functional class with reduction in congestive symptoms at 6 month follow up. In a subset of 7 patients that underwent CMR follow up, a mean reduction in RV end diastolic volume of 12% was observed (61).

#### Pre-procedural imaging

Dimensions for device fabrication are derived from CT (61). Sizing is performed using perimeter derived diameter. The SVC portion of the stent is sized at the superior cavo-atrial junction, and 1, 1.5, and 2 cm, above this level. The inferior stent portion is sized at the inferior cavo-atrial junction and above the hepatic vein ostium. RA length and relationship of the valve element with the TV are also assessed for fabrication. Caval diameters of 16–35 mm and RA length of 40–80 mm are feasible for fabrication. Fluoroscopic projections for device deployment are derived from CT.

#### Intra-procedural imaging

The device is deployed under fluoroscopic guidance. TEE may be used as an adjunct, however, is not mandatory. Right ventriculography and SVC angiography are performed to identify anatomic landmarks. The device is introduced via right femoral access and then deployed craniocaudally from the SVC, through the RA, and into the IVC. Radio-opaque device markers are used to guide device orientation. The device is re-sheathable up to 90% deployment. After deployment, right ventriculography and bicaval angiography are performed to evaluate continence of the valvular element and assess for endoleaks. Fusion imaging of echocardiographic and CT imaging with fluoroscopic projections has also been described to guide deployment (88, 89).

## Miscellaneous therapies

### Mistral

The Mistral device (Mitralix, Yok'neam, Israel) is a novel therapy for leaflet approximation and treatment of TR. The device consists of a spiral-shaped nitinol wire which achieves leaflet approximation through rotational grasping and approximation of the chordae tendinae. The device is delivered using an 8.5Fr steerable sheath via femoral venous access and is available in two sizes. Procedural imaging guidance is performed with fluoroscopy and TEE. After establishing access, the delivery catheter is advanced to the RA and the Mistral device is unsheathed to assume spiral configuration. The device is centred in the annulus, advanced into the RV, and maneuvered at the chordal level to the intended commissure for treatment. The device is rotated ∼5 times. Chordal engagement and device stability assessed with TEE and fluoroscopy. Evaluation for tricuspid stenosis and residual TR severity is performed before device release. Implant feasibility and reduction in TR has been demonstrated in the first in-human case series (90). The TRIBUTE—Pivotal study for the “Mistral” implant for the treatment of tricuspid/tricuspid valve leakage by percutaneous valve repair is currently underway (MOH_2021-06-09_010033).

### Tripair

The Tripair device (Coramaze technologies, Petah Tikva, Israel) is a transcatheter spacer device designed for treatment of functional TR. The device consists of a spacer balloon which crosses the tricuspid valve to seal gaps in leaflet coaptation attached to a self-expanding, crown shaped, right atrial frame which anchors the device in the right atrium. The device is delivered via an 18Fr steerable catheter via femoral venous access. The Tripair device is currently in pre-clinical testing (91).

### Croívalve DUO system

The Croívalve DUO Tricuspid Coaptation Valve system (Croívalve, Dublin, Ireland) is a novel transcatheter device for TV repair and replacement. The device is composed a coaptation valve which acts as a spacer to seal gaps in leaflet coaptation which contains a central valve to support diastolic flow. The device is anchored to a stent in the SVC via a support catheter which allows for respiratory and cardiac motion. The device is delivered via right internal jugular access. Procedural guidance is based on standard imaging with fluoroscopy and TEE (92). An early feasibility study of the efficacy and safety of the Croívalve system is currently underway (NCT05296148).

## Conclusion

In the absence of robust data supporting the use of isolated TV surgery for symptomatic severe TR, as well as the high in-hospital mortality associated with surgical intervention, transcatheter tricuspid valve therapies are rapidly evolving. Multiple classes of therapeutic devices are currently in development. Some devices have a surgical predicate with data to help define appropriate clinical and anatomic patient populations for these novel devices. Other devices are completely novel in their approach to disease pathophysiology. However, the future of all these devices will be in large part be determined by safety and efficacy, with the expectation of lower procedural morbidity and mortality and improvements in long-term outcomes. Multi-modality imaging is currently the cornerstone of patient selection, device choice and procedural success. Advanced imaging tools for analysis of anatomic as well as hemodynamic suitability for TTVI have also advanced our understanding of the pathophysiology of TR. Intraprocedural imaging software and devices have been integral to the procedural success of transcatheter tricuspid valve therapies and helps drive the innovation around device development.
